# Smart energy monitoring and power quality performance based evaluation of 100-kW grid tied PV system

**DOI:** 10.1016/j.heliyon.2023.e17274

**Published:** 2023-06-14

**Authors:** Lavanya A, Divya Navamani J, Geetha A, Viswanathan Ganesh, M. Jagabar Sathik, Vijayakumar K, Ravichandran D

**Affiliations:** a Department of Electrical and Electronics Engineering, SRM Institute of Science and Technology, Kattankulathur, India; b Department of Energy and Environment, Chalmers University of Technology, Gothenburg, Sweden; c Renewable Energy Lab, Prince Sultan University, Saudi Arabia

**Keywords:** Solar, 100-kW, PV, Power quality, Energy yield

## Abstract

Globally, the demand for energy from renewable sources is growing due to the increasing electricity consumption and the pollution of fossil fuels. The government has framed various policies to facilitate green energy generation, encouraging renewable energy source usage through PV installations in multiple sectors, including educational institutions. The primary objective of this paper is to propose a methodological approach for analysing the performance of the installed PV system on the rooftop of a university building in Tamil Nadu, India. The site selected is favourable for electricity generation from PV systems with an average global solar radiation of 5.82 kWh/m^2^day. Solar energy changes periodically with annual and daily variations and is not steady due to seasonal changes. The step-by-step performance assessment and the annual performance of the 100-kW solar PV system, which was instituted in 2019, with the forecasted parameters, are presented in this paper. Therefore, the assessment analysis is carried out in four phases: feasibility assessment, Energy yield assessment, Life cycle assessment, and Power quality assessment. To improve the solar PV output and efficiency, considering the solar irradiation, temperature, wind velocity, etc., PV yield is measured to evaluate the PV system's energy metrics. This paper also considers the carbon credits earned, solar power generated in the location, and the payback period. The power quality assessment is carried out in this paper to test the PV plant's compliance with effective grid integration.

## 1 Introduction

The irreversible environmental consequences of fossil fuels, like global warming and their escalating cost, increase the need for renewable energy sources [[1], [2], [3]]. Solar energy is non-polluting, available in nature, and costs less. Trillion and trillions of such power are received by earth and left unused India is the seventh-biggest nation in the world, having a size of 32.8 lakh sq. km. It receives about 4–7 kilo Watt-hour of solar energy per square metre per day with trillions of kilo Watt-hour per year of incident solar energy. Solar installed capacity was 47.7 GW as of October 30, 2021 in India. Therefore, India is endowed with an enormous amount of solar energy potential.

**Fig. 1:**
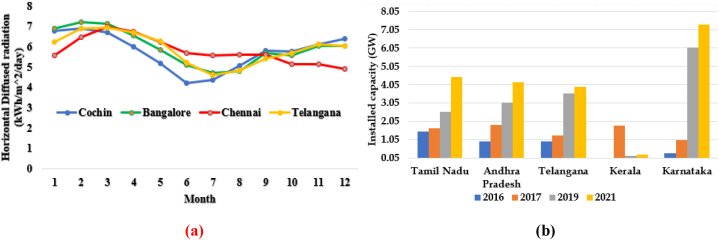
(a) Annual solar radiation in selected cities (b) Solar PV installed capacity in southern part of India.

Electric energy generation in PV cells is proportional to the prone area and the concentration of global insolation received [[4], [5], [6], [7]]. The PV resource availability in the southern cities like Cochin, Bangalore, Telangana and Chennai and the states Andhra Pradesh, Karnataka and Tamil Nadu in south India, along with the growth in PV installation in the past four years, is clearly shown in Fig. 1. The total installed cost of PV in India is around $793 per kilowatt, encouraging new PV projects. Therefore, it is becoming increasingly attractive and researchers are attracted to PV projects and their performance assessment [[8], [9], [10], [11], [12], [13], [14]]. The primary objective is to carry out a comprehensive and insightful analysis and overview of the installation and performance of the selected PV plant. In this paper, a 100-kW solar PV system is analysed in the climatic conditions of Kattankulathur, Tamil Nadu, India and its performance is presented. The collective solar installed capacity in the TN is 4316 MW and targets to install 8971 MW by 2022. In the past five years, the tremendous increase in solar PV installation shown in Fig. 1(b) further motivated this case study for research.

The development in power electronics and including power converters in the solar PV system, is vital for popularizing the PV generation system [[15], [16], [17], [18], [19], [20], [21], [22], [23], [24], [25]]. Therefore, power quality enhancement is the main challenge in power systems [26,27]. The following summarizes the findings of the literature review [[2], [3], [4], [5], [6], [7], [8], [9], [10], [11], [12], [13], [14], [15], [16], [17], [18], [19]]. A detailed investigation of both the pre-installation and post-installation performance metrics, like the geographical nature of the location, energy yield analysis, cost analysis, energy expenditure, etc., needs to be included. The research papers did not focus on a dedicated, intelligent monitoring system for long-term study and research. Software validation with ground data must be dealt with in detail in the literature. A rare discussion about the installed PV plant's energy payback and power quality analysis is found. This paper incorporates most of these factors, and accordingly, a single methodological approach is proposed.

## 2 Methodology

The methodology followed in this paper for smart energy monitoring and power quality performance-based evaluation of a 100-kW PV system is shown in the flowchart. The objective also includes the examination of the PV grid integration criterion, comprising voltage sag, voltage flicker, harmonics, voltage unbalance, and frequency fluctuations, and to confirm the high quality of the generated and delivered PV power.

### 2.1 Load details in study area

The load calculation is the first step in calculating the energy requirements and usage. Therefore, the design of the PV system is based on the load demand in that location. The load is estimated by enumerating the appliances' ratings and working hours. The overall average energy demand in watt-hours or kilowatt-hours daily and annually is also calculated. Table I shows estimated load details.

**Table I:** Load details.

S.No	Location	Lighting load	Fan load	UPS kVA	Motor HP
1	Strength of Material Lab	27	24	3 × 0.6 kVA	5HP-5, 2HP-2, 1HP-1
2	Metrology Lab	35	19	5 kVA	1HP-2, 0.5HP-5
3	Project Room	3	2		
4	FM Lab	23	31	6 kVA	2HP-2, 1HP-12
5	Passage Area	8	_		WATER COOLER-1
6	Rest Room	6	2		
7	Lab-1	13	8	20 kVA	
8	Lab-2	18	15	30 kVA	
9	Drawing Hall	18	15		
10	Lab-3	12	8		
11	Lab-4	10	7		
12	Rest Room	3	1		
	TOTAL	85	127	62.8 kVA	

**Fig. 2:**
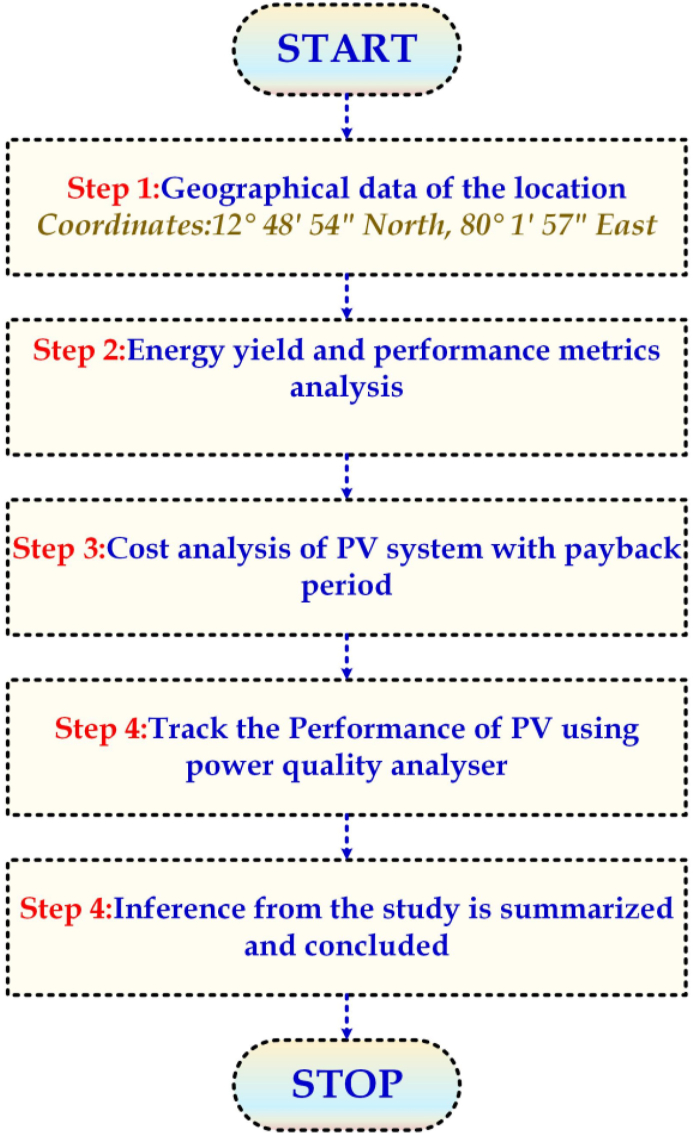
Flowchart of the methodology followed in this paper.

In the load distribution analysis, the ceiling fan and LED lighting loads are more significant than CFL lighting and other loads connected to the system. Since the case study is conducted in an educational institution, most are lighting and fan load. Next, after load estimation, the various steps followed in the PV design are shown in the flowchart below in Fig. 2.

## 3 PHASE-I

### 3.1 Geographical data of the location

Solar PV facility (100 kWp) is mounted on the roof of the Mechanical hanger block at SRM Institute of Science and Technology's Kattankulathur campus in Chennai, India.

This grid-tie PV system is made up of polycrystalline silicon (LE24P325) 325 kW PV panels, each measuring 156.75 × 156.75 mm and weighing 21.5 kg altogether. It contains 72 cells that are made of tempered glass and coated to reduce reflections. The anodized aluminium alloy frame has an overall thickness of 3.2 mm. Table II contains information on the PV plant.

**Table II:** PV Plant location and data.

Design parameter	Data
Plant Power	100 kWp
Location	Potheri, SRM Nagar, Kattankulathur, Tamil Nadu
Latitude and Longitude	12.49126°,80.0224°
Inverter model and Manufacturer	2 X RPI M50A, Delta Electronics India Pvt Ltd.
PV panel type	Multicrystalline
Panel Wattage	325 W
No. of PV Panels	320
Plant area	680 m^2^
Module Efficiency	16.72
Panel Tilt	9°

**Fig. 3:**
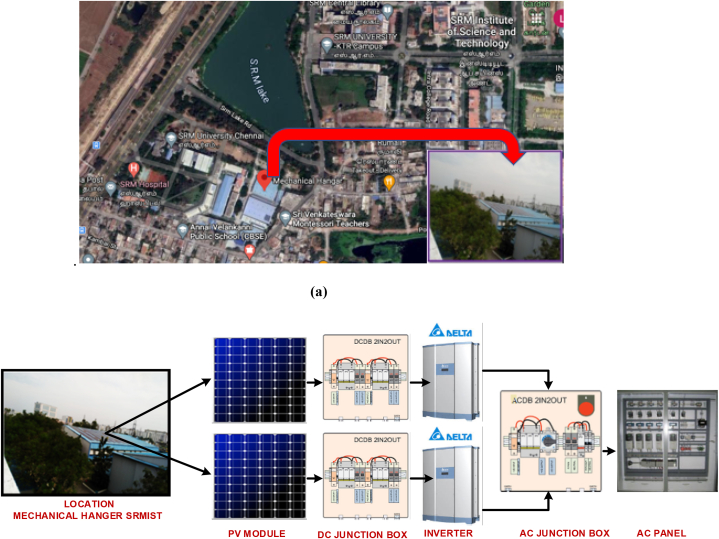
PV conversion, measurement and data monitoring system.

**Fig. 4:**
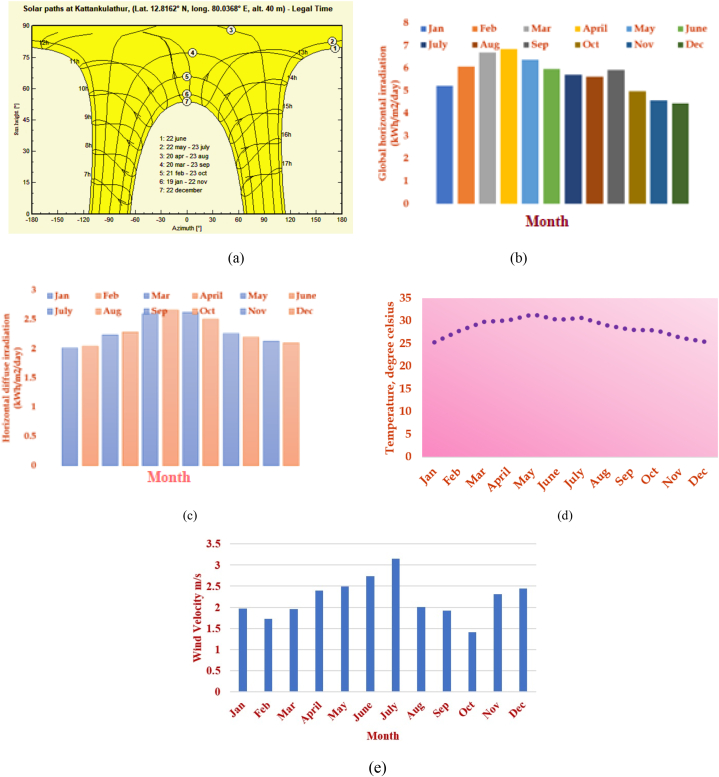
(a) Sun path diagram (b) Global horizontal irradiation in kWh/m^2^ (c) horizontal diffuse irradiation in kWh/m^2^/day - (12 months) (d) Monthly temperature variations (e) Wind velocity.

Fig. 3 depicts the data monitoring system set up in the exact location for monitoring and analysing the PV data generated and consumed. The PV module output is dc converted to ac output with the aid of the inverter arrangement and other balance of components. Fig. 4(a) shows the solar path for the specified latitude of 12.81°N, longitude of 80.03°E, and altitude of 40 m. The projection of the sun's path has a flat surface on top of the graph, from sunrise to sunset is shown with the sun height variation daily and monthly for significant sunlight values throughout the day depicted. Proper observation shows that the chart's most significant arc at the top is June 22, while the smallest is December 22, showing the location's average day length. The information about the altitude can be determined at a particular time in a specific month from this diagram.

The measured global solar irradiation and horizontal diffuse irradiation in kWh/m^2^/day for an entire year are portrayed in the graph shown in Fig. 4(b) and (c) in that particular location specified for 12 months. It is seen that from March to May, global radiation is at its peak and from May to July, horizontal diffuse irradiation is at its peak. This shows the monthly average daily solar radiation profile on the specified location. The annual temperature and wind velocity vary with different months throughout the study period for a specific area, as given below in Fig. 4(d) and (e). This helps to understand the location's temperature and wind velocity variation trend.

### 3.2 Description of installed PV system

The PV modules are stacked in two sets of ten parallel strings, with 16 PV modules in two stages in each string to generate a 100-kW rating. The complete PV module group is connected to a single Delta RPI-M50A grid-tie inverter through an inbuilt DC disconnect switch and feeds directly into the grid with two such inverters. The inverter's input and output appear on the front panel screen. It gives details such as DC and AC voltage and current, daily and monthly power and energy produced, and additional relevant data. Each group of ten strings is therefore connected to a grid-tie inverter.

Table III depicts the plant's electrical energy generated in one year. Peak energy generation occurred in April and May; the lowest was noticed in January. The initial Phase-I analysis is completed, and the following phase-II deals with the performance metrics analysis. The data acquired in phase I is utilized for the second performance analysis phase.

**Table III:** Solar energy generation from 100 kW plant in 2020.

Month 2020	kWh	Month 2020	kWh
Jan-20	3600	Jul-20	8766.32
Feb-20	4500	Aug-20	11799.41
Mar-20	5527.18	Sep-20	10882.95
Apr-20	14362.27	Oct-20	11204.96
May-20	14679.21	Nov-20	8739.39
Jun-20	12877.22	Dec-20	4020.31

## 4 PHASE-II

In this phase of analysis, the energy yield assessments are discussed to determine the energy possibly produced by the PV plant, which has a straight influence on the predicted bottom line of the issue.

### 4.1 Energy yield analysis

**Fig. 5:**
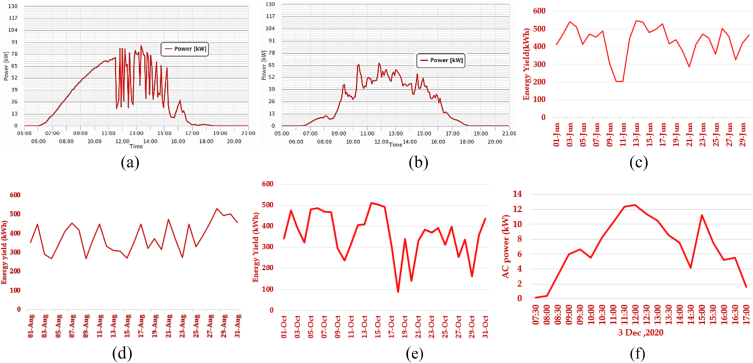
Inverter energy yield and AC power output (a) power generated in June 2020 (b) power generated in September 2020 (c) June month yield (d) August month yield (e) October month yield (f) AC power output on December 10, 2020 (Daily).

**Fig. 6:**
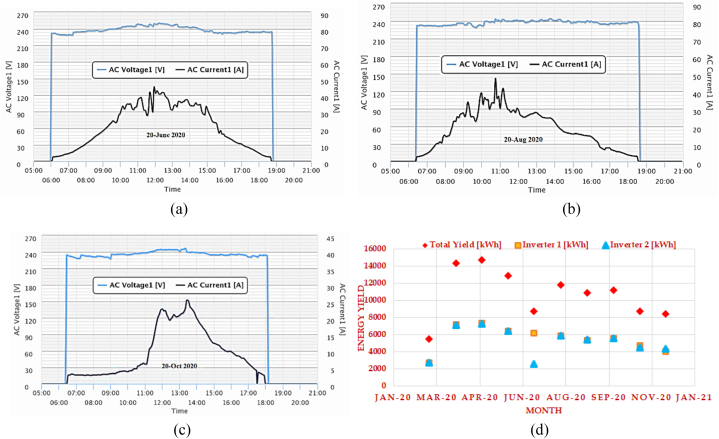
AC current and voltage, measured at various times throughout the year (a) June 20, 2020 (b) Aug 20, 2020 (c) October 20, 2020(d) Energy yield.

The energy yield from the PV system considered depends on a numeral factor which includes Solar radiation at the location, the slope of the PV unit, peak rated power of the PV modules, efficiency, effect of temperature on PV performance, shading due to trees and tall buildings, accumulation of dust and dirt and other related issues. Fig. 5(a)–(f) illustrates the trend in the variation of ac power generated and energy yield monthly and daily for June, August, September and October. Fig. 6(a)–(c) illustrates the changes in AC voltage and current based on the daily and monthly variation of solar radiation. Fig. 6(d) presents both the inverter's energy yield and the entire energy yield of the system in (kWh).

### 4.2 Performance indices calculation model

**Fig. 7:**
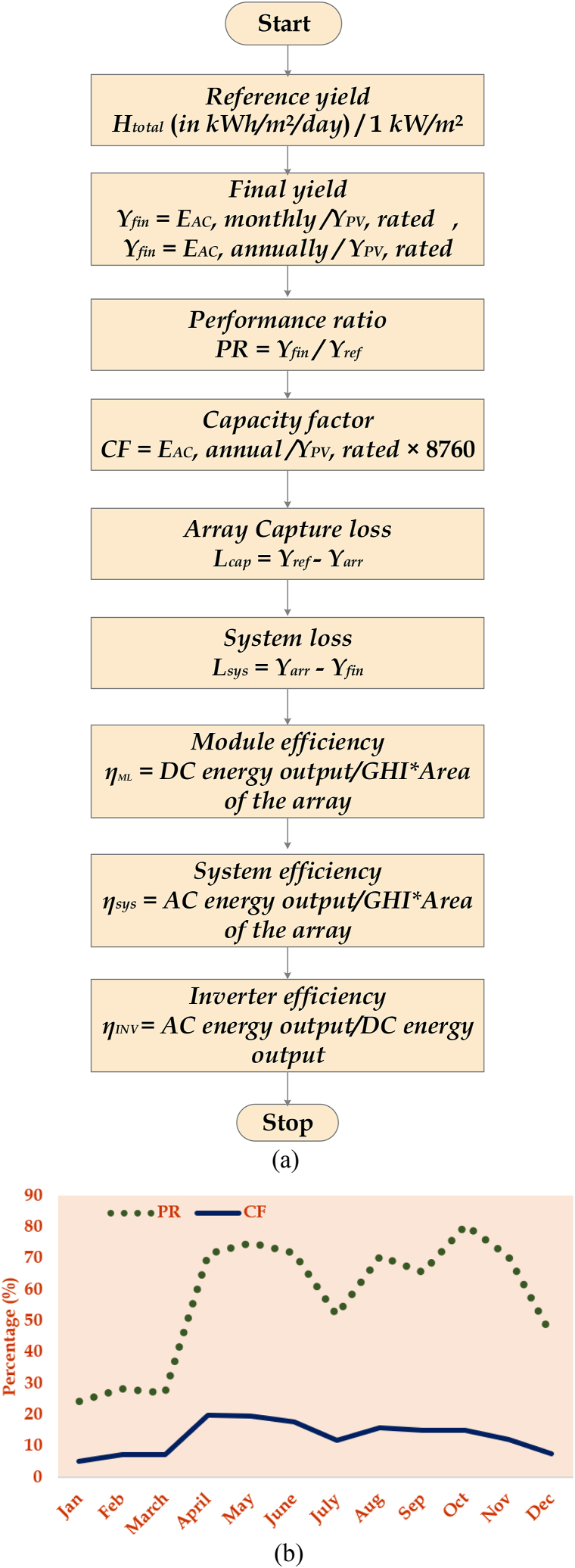
(a) Energy yield analysis modelling package (b) PR and CF comparison graph.

A portion of the power generated by the PV plant is used for internal power utilities. The power output varies daily due to variations in radiation. Because the PV plant's load does not vary during the day or night, the energy consumption remains constant. Due to rain or low radiation, the PV plant may stop generating power; therefore, the monitoring system logs every day's night consumption to analyse those conditions. Fig. 7(a) and (b) illustrates the energy yield analysis modelling and the Performance Ratio and Capacity Factor relationship.

This analysis is primarily executed to calculate the amount of energy the PV plant generates and analyse the system's performance. These criteria are essential for PV plant owners and consumers to decide on installing a PV plant. Due to technical issues, the plant performance in January and February recorded in the data monitoring system is not considered for the actual calculations of the plant. Table IV shows the values of the performance ratio and capacity factor calculated.

**Table IV:** Performance analysis of the 100-kW PV plant.

Month	Global horizontal irradiation (kWh/m^2^/mth)	Horizontal diffuse irradiation (kWh/m^2^/mth)	T Temp (°C)	E_y_ Energy Yield	S_y_ System yield	R_y_ Reference yield (H_t_/G_0_)	PR (%)	CF (%)
Jan	155.6	67.3	25.3	3942	37.9	155.6	24.3	5.2
Feb	164.3	58.4	27.8	4850	46.6	164.3	28.3	7.2
March	197.6	70.5	29.8	5540.31	53.2	197.6	26.9	7.4
April	193.5	79.8	30.1	14362.2	138	193.5	71.3	19.9
May	188.6	84.7	31.5	14679.6	141.1	188.6	74.8	19.7
June	172.8	84.9	30.3	12877.2	123.8	172.8	71.6	17.8
July	162.0	87.0	30.7	8766.3	84.2	162.0	51.9	11.7
Aug	160.7	92.8	29	11799.4	113.4	160.7	70.5	15.8
Sep	159.9	75.4	28.1	10882.9	104.6	159.9	65.4	15.1
Oct	133.9	73.7	27.9	11204.9	107.7	133.9	80.4	15
Nov	118.9	70.1	26.3	8739.3	84	118.9	70.6	12.1
Dec	118.9	64.4	25.4	5654.7	54.3	118.9	45.6	7.6
Year (Avg.)	160.5	75.7	28.5	9441.5	90.7	160.5	56.8	12.8

## 5 PHASE-III

Table V shows the energy generated in the PV array and grid. The various parameters considered in the analysis are PV array loss (Lc), System loss (Ls), reference yield Yr, final yield Yf, and Ya is the array yield. Lcr is represented to denote the array loss. Lsr is defined for the system loss. The calculated values of these parameters are given in Table VI.

**Table V:** PV radiation and power generation.

	Glob. Hor kWh/m^2^	Diff. Hor kWh/m^2^	T. Amp °C	Glob. Inc kWh/m^2^	Glob. Eff kWh/m^2^	E. array kWh	E. Grid kWh	PR
**January**	144.6	69.97	25.04	173.0	167.9	14,228	13,615	0.757
**February**	156.4	62.42	26.17	176.8	171.7	14,217	13,609	0.740
**March**	189.8	80.21	28.45	193.0	186.4	15,348	14,685	0.732
**April**	184.8	84.57	30.16	168.2	161.4	13,347	12,752	0.729
**May**	182.4	91.56	32.20	152.5	145.1	12,019	11,441	0.721
**June**	162.2	85.69	31.22	130.9	124.3	10,421	9884	0.726
**July**	157.9	85.94	30.66	129.7	123.4	10,381	9844	0.730
**August**	156.8	90.98	20.68	138.3	132.2	11,168	10,621	0.739
**September**	154.0	83.86	28.86	149.1	143.6	12,009	11,459	0.739
**October**	135.1	78.79	27.85	143.0	138.2	11,653	11,103	0.746
**November**	112.8	63.30	25.89	128,0	123.8	10,542	10,021	0.753
**December**	117.9	63.07	25.15	141.4	137.0	11,710	11,159	0.759
**Year**	1854.5	940.37	28.46	1823.9	1755.1	147,044	140,194	0.739

**Table VI:** Normalized performance coefficient.

	Yr kWh/m^2^/day	Lc	Ya kWh/kWp/day	La	Yf kWh/kWp/day	Lcr	Lsr	PR
**January**	5.58	1.167	4.41	0.190	4.22	0.209	0.034	0.757
**February**	6.31	1.432	4.88	0.209	4.67	0.227	0.033	0.740
**March**	6.22	1.464	4.76	0.205	4.55	0.235	0.033	0.732
**April**	5.61	1.329	4.28	0.191	4.09	0.237	0.034	0.729
**May**	4.92	1.192	3.73	0.179	3.55	0.242	0.036	0.721
**June**	4.36	1.023	3.34	0.172	3.17	0.234	0.039	0.726
**July**	4.19	0.965	3.22	0.167	3.05	0.231	0.040	0.730
**August**	4.46	0.996	3.46	0.169	3.29	0.223	0.038	0.739
**September**	4.97	1.121	3.85	0.176	3.67	0.226	0.035	0.739
**October**	4.61	0.999	3.61	0.171	3.44	0.217	0.037	0.746
**November**	4.27	0.888	3.38	0.167	3.21	0.208	0.039	0.753
**December**	4.56	0.928	3.63	0.171	3.46	0.203	0.037	0.759
**Year**	5.00	1.123	3.87	0.18	3.69	0.225	0.036	0.739

## 6 PHASE-IV

### 6.1 Life cycle assessment

The life cycle assessment technique is used to evaluate the environmental effect in various stages throughout the PV plant's existence in that location. This section provides a detailed assessment of the energy payback time and the economic analysis.

### 6.2 Energy payback time (EPT)

**Fig. 8:**
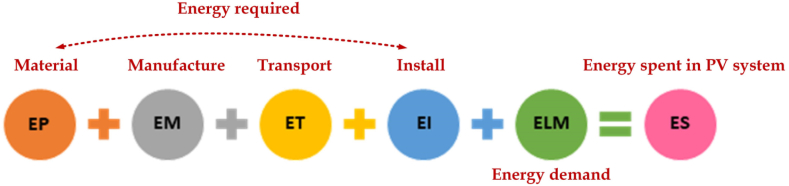
Energy expenditure equation.

The various parameters considered for analysis are total energy extracted from the material to produce the required energy from the PV system (EP), energy spent throughout the manufacturing process of the PV system (EM), energy consumed in the transportation of the materials throughout the lifetime (ET) of PV plant, energy spent while installing the system (EI) and energy demand management throughout the entire lifetime. Fig. 8 depicts the time required to compensate for the energy expended during PV system production when the total of all parameters mentioned above is divided by the annual energy produced.

All of the energy values given below were proposed by Tiwari et al. [28] and are viewed accordingly for the mathematical evaluation of these energy quantities. Equations (2), (3), (4), (5), (6), (7), (8), (9) are used for further analysis based on the values assumed for the various parameters taken into account for calculating total embodied energy per m^2^. The expression for embodied energy is given as

(1) $${Total\;embodied\;energy\left(E\right)=E}_{P}+E_{M}+E_{T}+E_{I}+E_{LM}$$

$$E=1513.59\;kWh/m^{2}$$

Overall area (A) of the 100-kW PV modules occupied is

(2) A = Total PV modules (325 Wattage) required × length × width

=320 × 1.96 m × 0.99 m = 620.92 m^2^

Total energy involved considering Eqs. (1) and (2) is

=1513.59 × 620.92 = 939.8 MWh

Energy yielded from the PV plant in one year = 113.29 MWh

Considering the above equations EPT is calculated as

(3) $$EPT=\frac{E_{P}+E_{M}+E_{T}+E_{I}+E_{LM}}{E_{AG}}$$

$$EPT=\frac{939.8}{113.29}=8.2\;yrs$$

EPT can be used to calculate the total performance of a PV system. Therefore, the Electricity Production Factor (EPF) can be calculated as given below.

(4) EPF = Output energy/Input energy

$$EPF=\frac{1}{EPT}=0.12$$

The energy yield of the 100 kW PV plant is used to get the capacity utilisation factor, which is given by

$$CUF=\frac{113.29\times 10^{3}}{100\times 8760}=0.12$$

### 6.3 Life cycle conversion efficiency

Life-cycle conversion efficiency analysis in view of various embodied energies is calculated over the life span of the PV system facility. The entire output of the plant is determined in terms of its solar output.

(5) $$\eta_{LCC}=\frac{E_{AG}\times L_{PV}-E_{TE}}{E_{SI}\times L_{PV}}$$

The PV plant is expected to last 30 years, and its annual total solar energy output is 1927 kWh/m^2^. Consequently, the efficiency of life cycle conversion is

$$=\frac{113.29\times 10^{3}\times 25-939.8\times 10^{3}}{1927\times 620.92\times 25}=0.063$$

### 6.4 Economic analysis

Economic analysis is discussed in this paper to recommend the economic prospects and increase the opportunities in promoting the PV system. The payback period signifies the time the money invested can be reimbursed and is calculated as given below.

• Basic payback period
• Lifecycle costing (LCC)

However, LCC estimate is more complex than a basic payback period in which the system cost and annual electricity output are evaluated.

#### 6.4.1 Basic payback period

The basic payback period calculation technique determines the time required to recoup the invested money from solar energy savings. Excluding the loss of value, interest rate, money value, operation and maintenance costs, and other lifetime costs of the PV system.

(6) $$Basic\;payback\;period=\frac{Capital\;investment\;of\;the\;100-kW\;PV\;system}{Yearly\;cost\;saving\;from\;solar\;energy}$$

The total energy produced in 2020 was 113.29 MWh. As per the Tamil Nadu electricity board, the tariff (kWh) for power consumption by private educational institutes and dormitories is seven rupees and fifty paise.

$$Basic\;payback\;period=\frac{24,44,000}{57.75e^{3}\times 7.50}=5.6\;years$$

The 100-kW PV plant installed is estimated to have around a five-year basic payback period, which gives the time to recoup the initial investment in establishing the PV system.

#### 6.4.2 Total cost of ownership

**Fig. 9:**
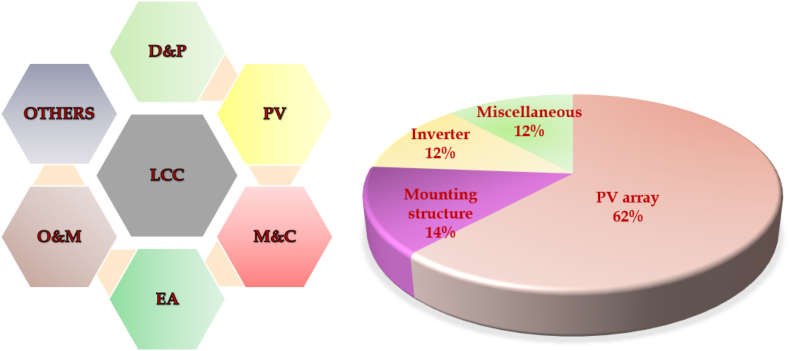
(a) Components of LCC (b) Breakdown of LCC in the location specified.

• The LCC model approach is built to assess the PV system during its life span. The PV plant's LCC includes the components like initial development and planning, PV module cost, electrical equipment, supporting structure and civil work. In addition to the operation and maintenance costs, miscellaneous and other expenses are included, as shown in Fig. 9. Lifecycle costing (LCC) includes Development and Planning (D&P), the cost of installing the PV system (PV), Mounting and civil (M&C), Electrical Apparatus (EA), Operation and Maintenance (O&M) and replacement costs.

India's rate of inflation is 5.1% (FY-2020) and rate of discount is set at 10%. The PV plant's lifetime is estimated to be 30 years. Table VII displays the total cost of ownership for a 100 kWp PV plant together with the Life Cycle Cost.

**Table VII:** Entire cost of ownership of 100-kW PV plant with 30 years lifespan.

Cost distribution	Cost incurred (Rs)	Present worth factor	Current worth (Rs)
PV modules	27,04,000/-	1	27,04,000/-
PV structure	6,24,000/-	1	6,24,000/-
DC-AC converter	5,20,000/-	1	5,20,000/-
DC-AC converter 10th year		0.634	1,64,480/-
DC-AC converter 20th year		0.402	1,04,520/-
Miscellaneous	5,20,000/-	1	5,20,000/-
OC 10th year		0.634	3,29,680/-
OC 20th year		0.402	2,09,040/-
Operation and Maintenance cost	41,600/-		
Operation and Maintenance cost recurring at year end		15.8	6,57,873/-
**LCC**			**39,09,593/-**

The current worth factor (CWF) of the investment is acquired by

(7) $$CWF={\left[\frac{1+i}{1+d}\right]}^{n}$$

where i = inflation rate (0.051), d = discount rate (0.1).

The cost will be reduced since the discount rate is considered higher than the inflation rate. The CWF factor reduces the product cost. The current value of the components that will require replacement after a particular period is calculated by evaluating the value of CWF. It is critical for calculating the current worth of future recurring investments by adding the cost of operation and maintenance of the PV plant over 30 years. It is determined as

(8) $${CWF}_{R@end}={k\left[\frac{1-k}{1-k}\right]}^{n}\;where\;k=\frac{1+i}{1+d}$$

For d = 0.1, k is equal to 0.955. K has a value between 0.95 and 1.05. In order to calculate the annual operating cost of the plant in terms of the current value of money, the annualised lifecycle cost (ALCC) of the PV system is evaluated. ALCC is derived from

(9) $$ALCC=\frac{LCC}{{k\left[\frac{1-k}{1-k}\right]}^{n}}$$

$$ALCC=\frac{39,09,593}{15.8}=2,47,442/-$$

The 100 kW PV plant's overall energy output since the installation date is displayed in Table VIII. Rs. 7.50/- tariff rate per unit (kWh) is used to compute the overall profit made. Similarly, the averted CO2 is calculated by 0.8 kg/kWh times the energy yield.

**Table VIII:** Benefits of 100 kW PV plant at the location specified.

Energy output from the date of commission	Returns until Dec 2020	CO_2_ reduced until Dec 2020	Diesel conserved until Dec 2020
104.49 MWh	8.35 lakhs	83.59 Ton	10.45 kL

#### 6.4.3 Power quality analysis

**Fig. 10:**
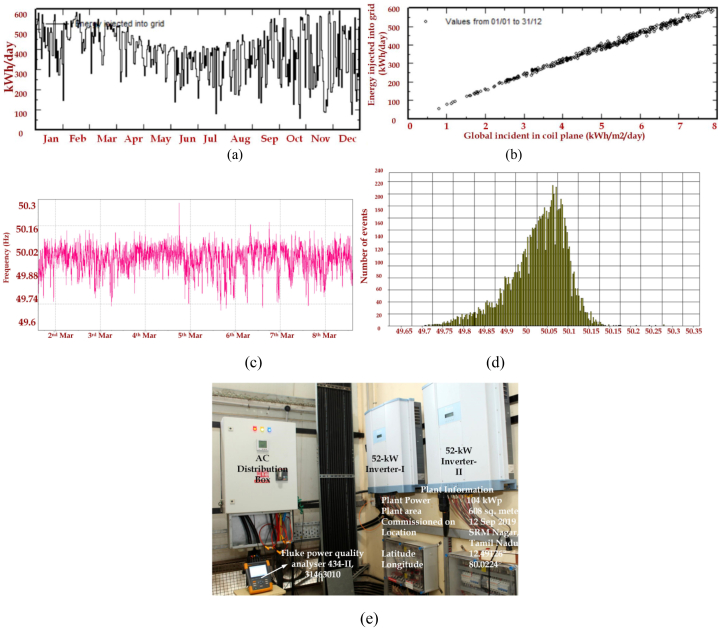
(a) Energy kWh/day (b) Energy injected into the grid (c) Frequency variation during March 2021 (d) Frequency variation with the events (e) Experimental setup for power quality assessment.

The data are recorded with Fluke power quality analyser 434-II, 31463010 in 1-min intervals for two weeks (14 days). The first recording was started on 26-03-2021, 10:47 and ended on 08-04-2021, 14:26. Fig. 10(a)–(e) presents the parameters analysed in the power quality analyser, the energy kWh/day, energy injected into the grid, frequency variation during March 2021, frequency variation with the events and the experimental setup for power quality assessment. Table IX presents the average power quality results obtained from the power quality analyser for three days.

**Table IX:** Average power quality results of the testing day.

Parameters	Phase	Mar 29, 2021	Mar 30, 2021	Mar 31, 2021
**Voltage (Phase to neutral) (V)**	V_rms(Ph-n) L1_	238.12	239.55	238.93
	V_rms(Ph-n) L2_	232.74	231.59	239.52
	V_rms(Ph-n) L3_	237.96	238.97	238.64
**Voltage (Phase to Phase) (V)**	V_rms(Ph-Ph) L12_	413.82	415.29	414.29
	V_rms(Ph-Ph) L23_	415.55	416.80	415.85
	V_rms(Ph-Ph) L31_	412.76	414.57	413.98
**Current (A)**	L1 (max)	101.6	99.5	95
	L2 (max)	101.4	99.3	94.9
	L3 (max)	102.8	100.8	96.2
**Frequency**	f (Hz)	49.99	50.02	50.03
**THD (voltage) (%)**	L1	2.66	2.52	2.03
	L2	2.30	2.42	1.87
	L3	2.36	2.30	1.92
**Active power (KW)**	L1	24.4	24.2	24.4
	L2	25	24.4	25
	L3	24.4	24.2	24.6
	Total	73.8	72.6	73.8
**Reactive power (KW)**	L1	1.6	1.6	1.4
	L2	2	2	2
	L3	2	1.8	1.8

Table IX shows the average power quality results during the testing days, including voltage, current, frequency, THD, and active and reactive power measured.

##### 6.4.3.1 Flickers

**Fig. 11:**
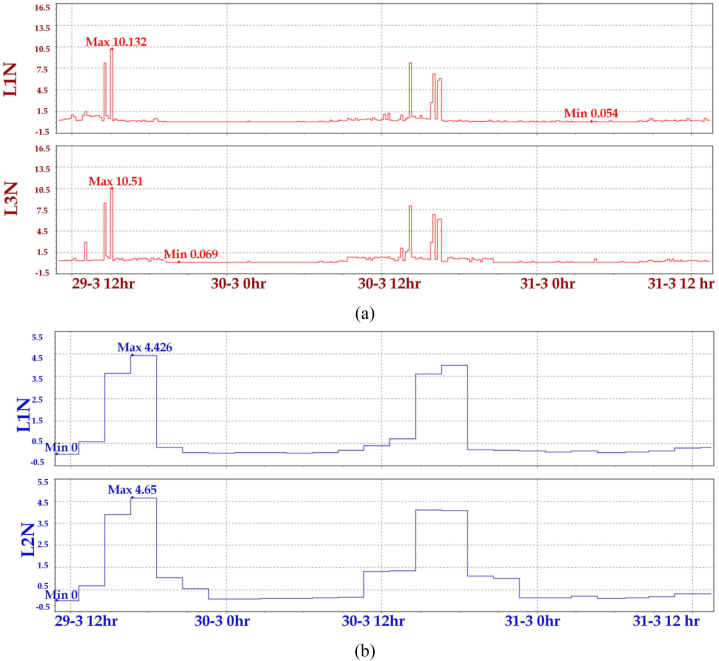
Flickers level (a) Short-term flickers (b) Long-term flickers.

**Fig. 12:**
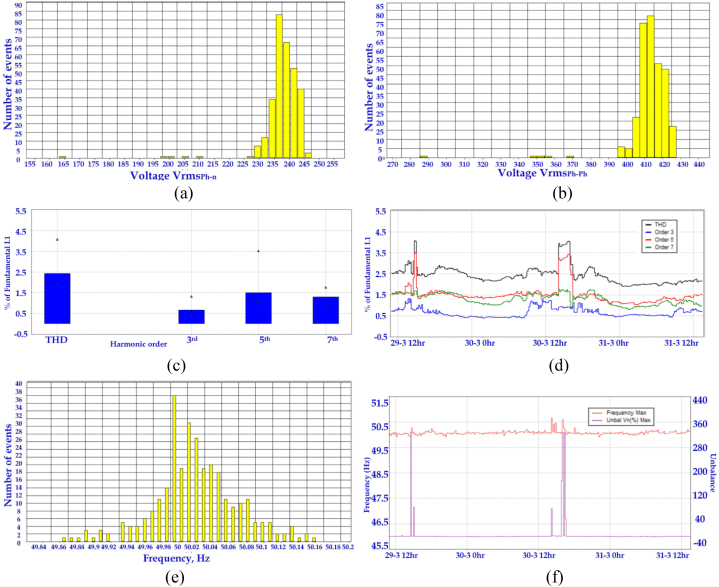
(a) Upper and lower extreme values of Vrms (Ph-n) (b) Upper and lower extreme values of Vrms (Ph-Ph) (c) Histogram display of harmonics (d) Harmonics time evolution plot (e) Upper and lower extreme values of frequency (f) Frequency/unbalance-time plot.

Flickers are introduced in the primary system due to voltage dip due to disconnection and connection of large loads. Abrupt and increased levels of flickers should be ignored. There are two types of flickers: PST and PLT. Short-term flickers (PST) are acquired every 10 min and long-term flickers (PLT) are calculated every 2 h, i.e., for 12 consecutive short-term flickers. The maximum and minimum value of PST and PLT is presented in Fig. 11(a) and (b). The significance of short-term and long-term severity is observed in this Fig. 11. The upper and lower extremes of RMS voltage (Phase to Neural and Phase to Phase) are presented in Fig. 12(a) and (b).

##### 6.4.3.2 Harmonics

Harmonics are periodic distortions in voltage and current waveforms due to non-linear loads. This harmonic will lead to overheating and even damage the equipment. In a power quality analyser, the harmonics can be obtained in histograms and time evolution. In Fig. 12(c) and (d), the total harmonic distortion (THD), 3rd, 5th and 7th harmonic order are presented for the study. THD is obtained with reference to the fundamental component. The non-linear impedance of the load creates harmonics in the current.

##### 6.4.3.3 Voltage unbalance

The term “unbalance” in three phase system refers to when the voltage and current have unequal magnitudes, and the phase angle between the components is not equal to 120°. The uneven load distribution in the phases leads to an unbalanced system. In a multiphase system, the load will run hotter if the voltage is not balanced. This reading is obtained by dividing the positive-sequence symmetrical components with negative-sequence components as per IEC 61000-4-30. Fig. 12(e) and (f) represents the Upper and lower extreme values of frequency and frequency/unbalanced voltage-time plot.

## 7 Results, discussion and future scope

This research will be expanded to assess the dynamic performance of the planned PV system under changing climate conditions. Furthermore, actions can be made to maximise the amount of electrical energy gathered. The 100-kW plant is outfitted with 320 325 W modules. It has a total surface area of 680 square metres. The analysis is conducted for a single assessment period (January to December 2020) to investigate the performance parameters of the 100-kW solar PV system. The suggested site's required meteorological data is gathered from PVsyst 7.1 software using NREL solar resource data. The worldwide horizontal solar irradiance is measured daily and weekly and contributes to the performance ratio calculation.

The study presents a location-specific contribution in the Kattankulathur area by supporting solar PV installation owners in identifying the kind of solar technology that is best laid for optimal production. The following discussions and observations are based on the results obtained during the plant performance analysis based on the conditions prevailing in the location during the assessment period.

Table V provides an illustration of the energy yield calculation for the plant with the performance ratio. The performance ratio is estimated to range from 76% in February to 26% in September based on this information. Similar to the previous parametric analysis, the capacity factor is determined for each month of the assessment period and it appears that it is elevated during March (19%) and low for September (7%). It is calculated what the average capacity factor will be in 2020. It is 12%, meaning that just 46 days out of the year the installed PV system can produce its entire amount of energy.

In March, the global horizontal irradiation (GHI) was 207 kWh/m^2^/month, while in December it was 138 kWh/m^2^/month. The chosen location's average GHI and temperature are roughly 173 kWh/m^2^ and 28.5 °C, respectively. In May, the average monthly temperature ranges from 31.5 °C to 25.3 °C. The energy yield is high in March, totalling 6928 kWh, and low in September, totalling 2428 kWh, according to the highest GHI.

Soiling, shading, temperature, module orientation, parasitic resistance, fill factor, cable thickness, and other factors all have an impact on power generation in PV systems.

Table X shows the overall energy generation from the 100 kW PV system since its installation. The entire profit is based on a price of Rs. 7.5/- per unit (kWh). Similarly, CO_2_ emissions are reduced. An optimal combination of PV-Wind-Battery systems can be implemented to supply electricity with optimum economic benefits.

**Table X:** Paybacks of installed 100 kW PV plant.

Total Energy yield (from the date of installation)	Total Revenue produced till Dec 2020	CO_2_ emission reduced till Dec 2020	Diesel energy saving till Dec 2020
114.8 MWh	9.18 lakhs	91.86 Ton	11.48 kL

Based on the research conducted in the selected PV plant, the following are the inference contributed through this proposed methodological approach for analysing the performance and extending a similar structure.

• The total energy produced by the facility in 2020 is 114.8 MWh.
• The greatest energy yield during the testing period was 6928 kWh in March 2020.
• The mean value of the performance ratio of the PV plant is 66%.
• The average capacity factor is 12%, which illustrates that the total energy output from the 100-kW plant is for 46 days in 2020.
• The payback time of the system's energy and electricity production factors is 8.2 years and 0.122, respectively.
• The system's energy is fed into the local grid and is not properly monitored.
• The Delta Electronics live monitoring system is used. But the energy delivered into the grid must be monitored and regulated regularly.

**Fig. 13:**
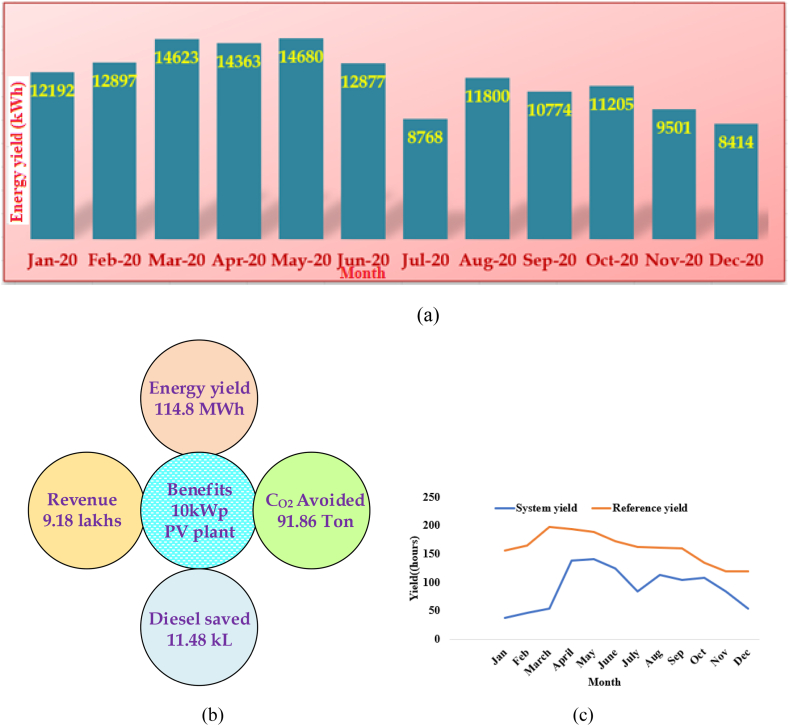
(a)Monthly energy yield in kWh during the year 2020 (b) Benefits of installed PV plant and (c) Yield analysis.

In general, March month has the longest reference yield, but the peak of the performance ratio is in winter in October. The performance ratio in summer is slightly lower with decreasing efficiency due to the drop in the module temperature rise. The forecasted electrical power from the proposed PV installation covers almost 60% of the electrical load in the location. This assessment reveals the energy yield, benefits of the PV plant investigated and the system yield shown in Fig. 13(a)–(c). Based on these findings the following suggestions are made.

1. Further extending the PV infrastructure and optimization will reduce the electricity expense of the university. This cost saved could be directed to research mainly in the field RESs.
2. This system has reduced pollution through clean energy power generation
3. PV system has embraced an eco-university environment.
4. The energy efficiency improvement manages the electricity demand and reduces CO_2_ emissions, significantly impacting the economy and environment. It contributes to achieving the vision of a green planet through sustainable development and zero-emission with sustainable green power generation.
5. This analysis and the conducted study contribute to the green building concept and achieve the sustainable development goals proposed globally. Every small step taken in this regard will result in making the planet a better place to live for the present and the future generation

In the future, the following few suggestions can be incorporated into the existing PV system, like Microinverter can be installed instead of a string inverter to overcome issues related to shading/bird poo, etc. Auto water spraying approach can be used to clean the PV panel by detecting the quantity of dust/dirt created in the PV panel on a regular basis. Panel orientation using a tracker with an actuator and controller is an option. Solar radiation measuring instruments and weather monitoring systems can be installed to follow the prevailing conditions and corresponding PV power generation data collection.

## 8 Conclusion

This manuscript studied and analysed a 100 kWp grid-connected PV system installed in the chosen location. The paper quantitively investigates the viability of installing a PV system on the campus rooftops, and the following conclusions are drawn from the evaluation. A 100 kW PV plant installed can reduce 91 tons of carbon dioxide emissions in one year. The diesel energy saving is about ten lakhs considering Rs. 91 in Chennai, India. Diesel consists of 86% of carbon dioxide. This system has saved 11.8 kL of diesel in two years, reducing carbon dioxide emissions. The resultant PV energy generation and the solar radiation input are monitored and used to calculate the performance parameters. The outcome obtained is related to one whole year of operation of the PV system in the chosen location. The average Performance ratio of the PV system is calculated as 66%. A basic payback period of 5.6 years can be obtained based on the performance evaluation, which gives the time to recover the initial investment in establishing the PV system.

The following observations are made in this evaluation, most of the PV energy generated by the 100 kW PV plant is expended on the university campus. Still, the PV system sometimes generates power without consumption or shuts down during holidays, including 15 days of government holidays. The unused PV power generated can be stored or diverted to neighbouring loads during this period. The power quality analyser was installed at the 100 kWp grid-connected PV system to measure the RMS voltage, flickers, Total harmonic Distortion, voltage unbalance and frequency for two weeks.

No significant research projects are currently conducted to study the PV system installed in the chosen location. Further, the solar radiation data collection and review of the operation periodically and monitoring of the system's proper working still need to be done. Therefore, intensive research is required to study and improve the system's performance, effective utilization, and expansion in the future.

## Author contribution statement

A. Lavanya - conceived and designed the experiments; performed the experiments; wrote the paper.

J. Divya Navamani - analysed and interpreted the data; wrote the paper.

Geetha. A - contributed reagents, materials, analysis tools or data; wrote the paper.

Viswanathan Ganesh; Jagabar Sathik; D. Ravichandran - conceived and designed the experiments; wrote the paper.

Vijayakumar. K - conceived and designed the experiments, contributed reagents, materials, analysis tools or data; wrote the paper.

## Data availability statement

Data will be made available on request.

## Declaration of competing interest

The authors declare that they have no known competing financial interests or personal relationships that could have appeared to influence the work reported in this paper.
